# The Genome of the Netherlands: design, and project goals

**DOI:** 10.1038/ejhg.2013.118

**Published:** 2013-05-29

**Authors:** Dorret I Boomsma, Cisca Wijmenga, Eline P Slagboom, Morris A Swertz, Lennart C Karssen, Abdel Abdellaoui, Kai Ye, Victor Guryev, Martijn Vermaat, Freerk van Dijk, Laurent C Francioli, Jouke Jan Hottenga, Jeroen F J Laros, Qibin Li, Yingrui Li, Hongzhi Cao, Ruoyan Chen, Yuanping Du, Ning Li, Sujie Cao, Jessica van Setten, Androniki Menelaou, Sara L Pulit, Jayne Y Hehir-Kwa, Marian Beekman, Clara C Elbers, Heorhiy Byelas, Anton J M de Craen, Patrick Deelen, Martijn Dijkstra, Johan T den Dunnen, Peter de Knijff, Jeanine Houwing-Duistermaat, Vyacheslav Koval, Karol Estrada, Albert Hofman, Alexandros Kanterakis, David van Enckevort, Hailiang Mai, Mathijs Kattenberg, Elisabeth M van Leeuwen, Pieter B T Neerincx, Ben Oostra, Fernanodo Rivadeneira, Eka H D Suchiman, Andre G Uitterlinden, Gonneke Willemsen, Bruce H Wolffenbuttel, Jun Wang, Paul I W de Bakker, Gert-Jan van Ommen, Cornelia M van Duijn

**Affiliations:** 1 Department of Biological Psychology, VU University Amsterdam, Netherlands Twin Register, Amsterdam, The Netherlands; 2 University of Groningen, University Medical Center Groningen, Department of Genetics, Groningen, The Netherlands; 3 Molecular Epidemiology Section, Leiden University Medical Center, Netherlands Consortium for Healthy Ageing, Leiden, The Netherlands; 4 Department of Epidemiology, Erasmus Medical Center, Rotterdam, The Netherlands; 5 European Research Institute for the Biology of Ageing, University Medical Center Groningen, University of Groningen, Groningen, The Netherlands; 6 Hubrecht Institute, Royal Netherlands Academy of Arts and Sciences, University Medical Center Utrecht, Utrecht, The Netherlands; 7 Netherlands Bioinformatics Centre, Nijmegen, The Netherlands; 8 Department of Human Genetics, Center for Human and Clinical Genetics, Leiden University Medical Center, Leiden, The Netherlands; 9 Leiden Genome Technology Center, Leiden University Medical Center, Leiden, The Netherlands; 10 Department of Medical Genetics, University Medical Center Utrecht, Utrecht, The Netherlands; 11 BGI-Shenzhen, Shenzhen, China; 12 BGI-Europe, Copenhagen, Denmark; 13 Department of Biology, University of Copenhagen, Copenhagen, Denmark; 14 The Novo Nordisk Foundation Center for Basic Metabolic Research, University of Copenhagen, Copenhagen, Denmark; 15 Department of Human Genetics, Radboud University Medical Centre, Nijmegen, The Netherlands; 16 Department of Gerontology and Geriatrics, Leiden University Medical Centre, Leiden, The Netherlands; 17 Department of Medical Statistics and Bioinformatics, Leiden University Medical Centre, Leiden, The Netherlands; 18 Erasmus Medical Centre, Genetic Laboratory Internal Medicine, Rotterdam, The Netherlands; 19 Department of Clinical Genetics, Erasmus University Medical School, Rotterdam, The Netherlands; 20 LifeLines Cohort Study & Department of Endocrinology, University Medical Center Groningen, University of Groningen, Groningen, The Netherlands; 21 Department of Human Genetics, Leiden University Medical Centre, Leiden, The Netherlands

**Keywords:** whole-genome sequence, trio-design, population genetics

## Abstract

Within the Netherlands a national network of biobanks has been established (Biobanking and Biomolecular Research Infrastructure-Netherlands (BBMRI-NL)) as a national node of the European BBMRI. One of the aims of BBMRI-NL is to enrich biobanks with different types of molecular and phenotype data. Here, we describe the Genome of the Netherlands (GoNL), one of the projects within BBMRI-NL. GoNL is a whole-genome-sequencing project in a representative sample consisting of 250 trio-families from all provinces in the Netherlands, which aims to characterize DNA sequence variation in the Dutch population. The parent–offspring trios include adult individuals ranging in age from 19 to 87 years (mean=53 years; SD=16 years) from birth cohorts 1910–1994. Sequencing was done on blood-derived DNA from uncultured cells and accomplished coverage was 14–15x. The family-based design represents a unique resource to assess the frequency of regional variants, accurately reconstruct haplotypes by family-based phasing, characterize short indels and complex structural variants, and establish the rate of *de novo* mutational events. GoNL will also serve as a reference panel for imputation in the available genome-wide association studies in Dutch and other cohorts to refine association signals and uncover population-specific variants. GoNL will create a catalog of human genetic variation in this sample that is uniquely characterized with respect to micro-geographic location and a wide range of phenotypes. The resource will be made available to the research and medical community to guide the interpretation of sequencing projects. The present paper summarizes the global characteristics of the project.

## Introduction

The last decade has seen rapid developments and breakthroughs in unraveling the genetic etiology of complex genetic disorders.^1^ Genome-wide association studies (GWAS) have uncovered thousands of variants influencing major diseases and complex human traits, including cardiovascular disease, diabetes, dementia, schizophrenia, height, and body mass index (BMI). Despite major successes in gene discovery, for many traits the percentage of variance explained by GWAS loci is relatively low, leaving a substantial part of the variation to be explained. Approaches that estimate genetic variance based on a large series of GWAS variants such as GCTA^2, 3^ show that aggregate effects of genome-wide significant common variants and those that do not reach this criterion explain substantially larger amounts of variance for, for example, human height,^4^ major depression^5^ or type 1 and 2 diabetes.^6^ Clearly, GWAS have not yet reached their limits, as is demonstrated by the many new loci detected after increasing sample sizes.

The use of dedicated arrays such as the metaboChip and immunoChip, which include new common and rare variants from the established GWAS regions, confirm the findings of modeling studies that the heritability not yet explained is to be found in a mixture of unmapped and untagged common, and not yet discovered rare variants.^7, 8, 9, 10^ Despite progress, our knowledge of the regions identified by GWAS is still limited. Considering that even for ‘simple' genetic diseases such as cystic fibrosis thousands of relevant mutations have occurred in a single gene, there is a need for in-depth sequencing and characterization of the genomic regions identified thus far. In a selected, relatively small, group of 256 Sardinians with extreme LDL-C values who were resequenced and reimputed to the 1000 Genomes Project reference at six GWAS loci, the variance explained for blood lipid levels at known loci doubled through the identification of both rare and common variants.^11^

Thus, identifying low frequency and rare variants by resequencing will contribute to resolving the missing heritability. As rarer variants are more likely to be population specific,^12^ sequencing across a range of different populations is mandatory for translation into public health and clinical benefits. One aim of the 1000 Genomes Project is to create maps of genetic variation across multiple populations, but the number of individuals per population is modest. Tennessen *et al*^12^ identified >500 000 single-nucleotide variants (SNVs) in a sequencing project of 15 585 human protein-coding genes. The major part of these SNVs were rare (86% with a minor allele frequency<0.5%), unknown (82%) and population-specific (82%). A deep-sequencing study^13^ of 202 putative drug targets confirmed that most genetic variants occur at very low frequencies. To characterize a population, it is crucial to sequence as many individuals as possible to maximize the probability of capturing rare variants.

Findings from sequencing studies are also important for clinical applications and public health genetics. The interpretation of the ‘potential causality' of a novel variant after it has been identified in patients in a clinical setting, requires knowledge of its frequency in the population the patient belonged to. In view of its potential research and clinical applications, the Genome of the Netherlands (GoNL; http://www.nlgenome.nl) project was initiated as a part of BBMRI-NL^14^ (Biobanking and Biomolecular Research Infrastructure-Netherlands). The aim of GoNL was to sequence 1000 independent genomes from the Dutch population. These 1000 Genomes come from a representative sample of 250 parent-pairs with 1 or 2 offspring. Sequencing was done using genomic DNA to an intended average depth of 12x for each sample. Here, we describe the design of GoNL within the demographic setting of the Netherlands, the sampling frame and basic characteristics of the participants.

Geographically, the Netherlands is a small country of about 41 500 km^2^. Yet, the Dutch population of nearly 17 million inhabitants constitutes a middle-sized population within Europe and represents 0.24 percent of the world's total population. The Netherlands were populated by different groups.^15, 16^ Regional isolation and religious endogamy, as well as rapid population growth had key roles in shaping the population genetics of the Dutch. Population growth was low until the 19th century, and doubled between 1900 and 1950 from 5.1 to 10 million people and between 1951 and 2000 to 15.9 million. In 1901, the total fertility rate was 4.53, in 1950 it was 3.10 and in 2000 it had decreased to 1.72. From the 1970s onwards (http://statline.cbs.nl/StatWeb/publication), the number of non-western immigrants increased from ∼1.2% of the population to 10.4% in 2005 and is now stable at 11.6%. Immigrants have been of various genetic backgrounds, with Turkish and Moroccan origin being the largest groups followed by those of Asian and African origin from (former) Dutch overseas areas (Netherlands Antilles, Suriname and Indonesia). At the design stage of the GoNL project, we decided to include participants whose ancestors are born in the Netherlands. Sequencing of other groups was not possible without compromising the power for all the groups. However, extending sequencing beyond Caucasians remains a desirable aim.

**Figure 1:**
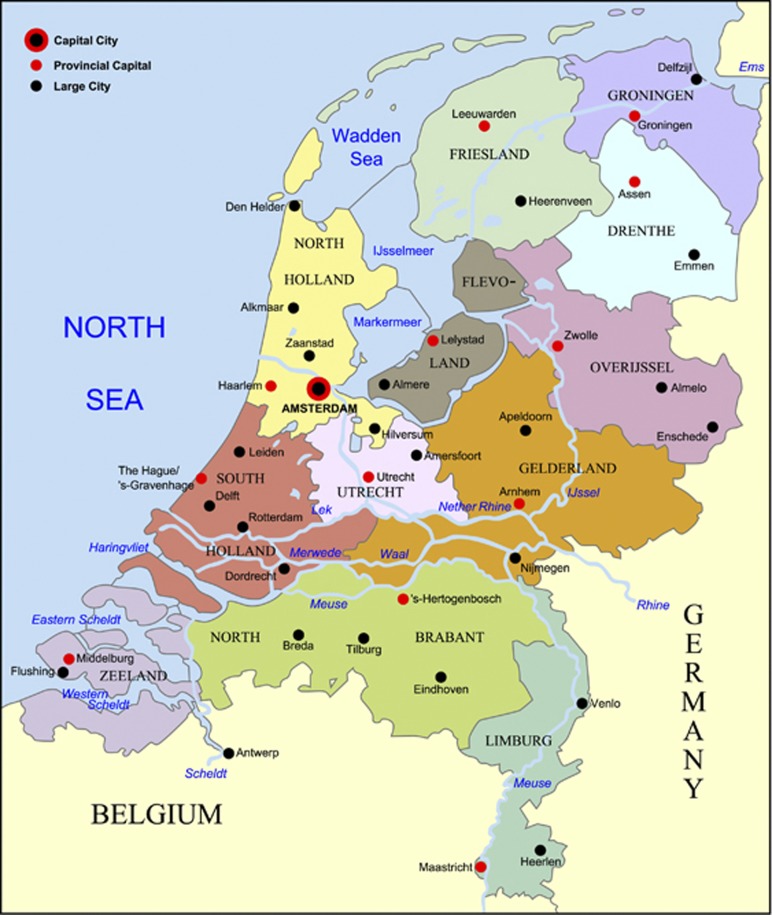
The 12 provinces of the Netherlands, the 12th province (Flevoland) is a recent province (land reclaimed from water) and was not included as a separate sampling unit (Image by Wikimedia Commons user Alphathon).

Another key decision concerned the selection of subjects over geographic regions. The Netherlands are historically divided into provinces, which are geographical units with their own governance dating back from the period that the Netherlands were a republic (1588–1795). Geographically, the distribution of the population in the Netherlands is heavily skewed with the majority (75%) of the population living in the Western and Middle provinces of the country including North-Holland (16% of the population including the capital Amsterdam), South-Holland (22% including the largest European harbor, Rotterdam), North-Brabant (15%, bordering Belgium, formerly part of the Dutch kingdom), Gelderland (12%, bordering Germany) and Utrecht (10% see Table 1 and Figure 1); only a small fraction lives in the Northern and Southern provinces. There is considerable evidence for regional differences in genetic make-up, for example, there is a significant North–South gradient both in height and eye color.^15^ Also studies of the Y-chromosome clearly pinpoint differences in genetic origin.

Representative sampling from the provinces of the Netherlands according to sample size would lead to major differences in numbers of subjects sequenced per region. Although this reflects the size and distribution of the population, many population genetic analyses are influenced by sample size, including linkage disequilibrium and founder effects. We, therefore, chose to include an approximately equal representation from the original 11 Dutch provinces (see Table 1), oversampling the two major cities Amsterdam and Rotterdam. The 12th province, which was established in 1986, was not included, as it was populated by recent immigration from surrounding areas.

### Trio design

A key characteristic of GoNL is its trio design. The sample unit consists of two parents and one offspring yielding information on four genomes (two per parent). There were three considerations that led to the choice of the trio design for GoNL despite the fact that a trio design reduces the effective sample size by one-third compared with a design that includes only unrelated individuals. First, a major aim is to provide a facility for imputation, rapid and valid haplotyping, and the trio design allowed GoNL to reduce the computational burden of haplotyping. Second, a trio design would enable reducing the number of false-positive variant calls by screening for violation of Mendelian segregation in the trio-aware calling of genotypes. Third, the trio design would allow for a characterization of new mutations within the Dutch population, in that novel variants that occur in offspring but not in parents can be assessed. This latter approach requires a separate calling and storage of variants in which non-Mendelian transmission is allowed. Our sequencing at intermediate coverage was considered to be optimal to address these aims.

### Selection of samples

In the Netherlands, several large-scale collaborative Biobank consortia have been established, that bring together patient samples and population-based collections of biological samples. GoNL is part of the Dutch branch of BBMRI (http://www.bbmri.nl/). In BBMRI-NL,^14^ ∼180 biobanks from all over the Netherlands have been united, including over 600 000 subjects with biological samples and phenotype information. For 100 000 to 150 000 subjects genome-wide SNP data are available, or are currently being generated. For the GoNL trio study, four population-based biobanks provided samples:

1. LifeLines (http://lifelines.nl/lifelines-research/general), which is a three-generation population-based cohort now including more than 100 000 participants (target 160 000) representing ∼10% of the northern part of the Netherlands.^17^
2. The Leiden Longevity Study (http://www.healthy-ageing.nl; http://www.langleven.net), which includes 3500 persons from families in which at least two long-lived siblings were alive and their parents were of Caucasian descent.^18^
3. The Netherlands Twin Registry (NTR: http://www.tweelingenregister.org), which ascertains two- and three-generation Dutch families from the entire country based on the presence of twins or higher order multiples in the family and includes nearly 180 000 participants.^19, 20^ The NTR Biobank collected biological samples in >10 000 participants.^21^
4. The Rotterdam studies, which consist of a population-based long-term follow-up study including 11 800 persons from Rotterdam^22^ and its surrounding area (http://www.erasmus-epidemiology.nl/rotterdamstudy) and the Genetic Research in Isolated Populations program (GRIP: http://www.epib.nl/research/geneticepi/research.html#gip) targeting the south western part of the Province North-Brabant.

**Figure 2:**
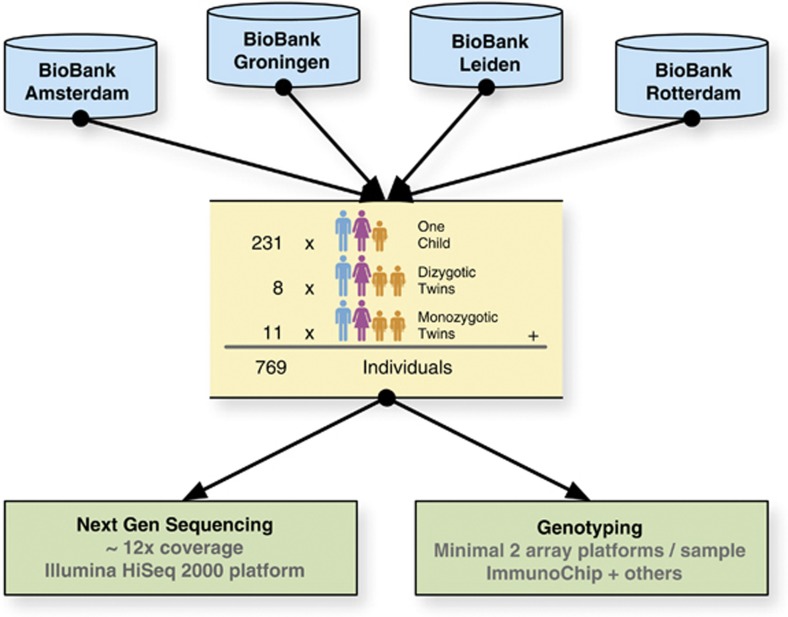
Sampling schedule for the GoNL: four population-based biobanks contributed samples for sequencing at the BGI (Beijing Genomics Institute).

Table 1 summarizes the geographical distribution of the GoNL participants over the Netherlands. A total of 769 subjects were included from 250 families. In 19 of the 250 families, 2 offspring were sequenced, 11 monozygotic (MZ) and 8 dizyotic (DZ) twin pairs (see Figure 2). All participants are part of active biobanks. For the majority of participants information on their place of birth and place of birth of their (grand) parents is known. As the participants come from prospective studies, a wealth of phenotype data is available, including risk factors for morbidity and mortality, and information on lifestyle and medication use. As part of BBMRI-NL, these data will be further enriched with metabolomics (NMR, mass spectrometry), whole-genome epigenetic (450 K Illumina methylation arrays) and RNA-sequencing data. Information on morbidity is collected by record linkage projects in the Netherlands with databases such as PALGA (The nationwide network and registry of histo- and cytopathology in the Netherlands), NKR (Netherlands Cancer Registration) and others.

Table 2 provides the demographic background of the sample. The mean maternal age at blood draw was 62 years (SD 8.4 years), the mean paternal age 64 (SD 8.6 years) and that of the offspring 36 (SD 9.3 years). On average there is a 5 cm increase in height in the second generation, as is consistent with general population data. Average BMI values show the population to be relatively healthy, although persons with severe obesity are also observed. Lipid levels are relatively low, in line with the low BMI and the relatively high percentage of lipid lowering medication users in the Dutch population.

### Sequencing

A coverage of 12x in both parents and the offspring was chosen based on balancing sensitivity (calling completeness) and accuracy (erroneous false-positive variants), within budgetary limits.^23^ This design gives 500 haplotypes at reasonable depth (that is, those transmitted to the children) and 500 haplotypes at half of that depth (that is, those not transmitted to the children). This design should significantly contribute to the globally existing information, as, for example, the 1000 Genomes Project has a 4x coverage. Paired-end sequencing of genomic DNA was done on the Illumina HiSeq 2000 platform (Illumina Inc., San Diego, CA, USA) at the Beijing Genomics Institute (BGI), starting in October 2010 and finished in June of 2011. Additionally, for each sample micro-array SNP data (Immunochip on all samples and at least one other GWAS array per sample) were generated by the biobanks. Sequencing was performed on 2–3 DNA libraries per sample which were run on 2–4 lanes of HiSeq 2000. The raw reads were subsequently analyzed following 1000 Genomes best practices, in collaboration with the Broad Institute and BGI. When reads were aligned against build 37 of the human genome coverage was, as expected, randomly Poisson distributed over the genome with a coverage peak in between 14–15x. The reads were cleaned by indel realignment, duplicate read marking and quality score recalibration, and SNP variants were called with a trio-aware caller. In parallel, a wide variety of short indels and complex structural variants were called and the SNPs were phased for imputation. Data processing involved 200 000 h of computation and was run on the Dutch national Target and BigGrid compute facilities supported by CIT/Groningen and SARA/Amsterdam. This effort resulted in 60 TB of aligned reads and variant calls (http://www.rug.nl/target/infrastructuur, http://www.biggrid.nl/). While the sequencing technology and data processing pipelines are being improved continually, variant detection, including short indels and complex structural variants in low-complexity regions continue to pose a challenge. The GoNL data analysis group applied Genome Analysis Toolkit (GATK) BreakDancer, Pindel,^24^ GenomeSTRiP, CNVnator, 123SV and DWAC-seq to discover and genotype diverse types of variants such as SNPs, insertions, deletions, tandem duplications, inversions and translocations.^25^ Each of these tools showed its own variation detection characteristics, and the joint use of the tools turned out most powerful. Detailed analytical descriptions of SNP and SV calling, and *de novo* and loss-of-function variants will be reported separately. Detection of *de novo* mutations and accurate genotyping from pedigree data is challenging, especially at low or intermediate depth of coverage. Both require accurate and quantitative calibration of the evidence supporting the individuals' genotypes using all the available information, including their familial relationship and population allele frequency.

### Genomic structure of the Netherlands

**Figure 3:**
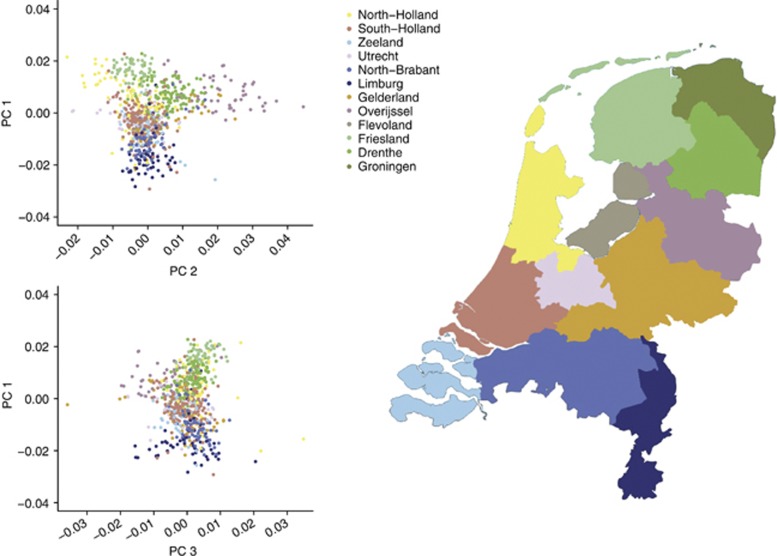
PCA results highlighting differences in genetic make-up across the Netherlands: the plots give PC1 *versus* PC2, and PC1 *versus* PC3.

To describe the regional genomic differences in the genetic make-up across the Netherlands, principal component analysis (PCA) was run using the EIGENSOFT package.^26^ The PCA was first run on 4441 unrelated Dutch individuals^15^ typed on the Affymetrix Human Genome-Wide SNP 6.0 Array (Affymetrix Inc., Santa Clara, CA, USA), with a SNP set excluding 24 long-range LD regions^27^ and LD-based SNP pruning (based on a variance inflation factor of 2), resulting in a set of 130 248 SNPs. These PCs were then projected on the 769 GoNL samples (using SNP calls release 2). Figure 3 summarizes the PC 1 *versus* PC 2 and the PC 1 *versus* PC 3 plots. Three PCs correlated significantly with geographic location and distinguished between: (1) the North and South of the Netherlands; (2) between the East and West; and (3) between the middle-band of the Netherlands and the rest of the country. When projected on the GoNL individuals, for whom we have the province of the current living address available, it is clear from Figure 3, that PCs capture the geographical variation in the data very well.

## Conclusion

The goal of GoNL is to establish a reference set of variants in the Dutch human genome that have a frequency of at least 0.5% by whole-genome sequencing of 1000 independent genomes. Our pilot analyses reveal that new variants are likely to emerge compared with 1000 Genomes and may thus contribute to sequence data acquired by others. The GoNL project includes a number of unique aspects compared with other ongoing projects (see Table 3). The sequencing of GoNL was based on DNA isolated from blood (untransformed cells), whereas sequencing in 1000 Genomes involved DNA derived from transformed lymphoblastoid cell lines. Second, the embedding of the study in population-based prospective epidemiological cohorts enables research of clinical outcomes associated with mutations. Third, the trio design of the project in combination with the medium coverage of each genome allows estimation of haplotypes accurately, and to call a substantial amount of the more rare and *de novo* variation (both SNPs and structural variants). For 19 families, additional monzygotic or dizygotic twin siblings were sequenced, which allows in-depth research targeting new variants. Finally, the additional data on clinical phenotypes, epigenetics, RNA sequencing and the metabolome of the sequenced individuals will allow functional studies of new variants.

One of the main purposes is to catalog both common and rare variants, and to use the Dutch reference genome as a basis to computationally infer the corresponding information in a much larger data set of 100–150 thousand Dutch GWAS samples that have been generated in the Dutch population. The creation of an imputation pipeline is an important deliverable of GoNL. An imputation pipeline was created on the European Grid network for cohorts participating in BBMRI-NL, which can also be used by cohorts from elsewhere. The pipeline requires cohorts to upload their imputation-ready genotype data. Next, the process aligns the data with the Dutch and/or combined reference set and imputation will be done following recent protocols like the ones for the GIANT (the Genetic Investigation of ANthropometric Traits) consortium, using parallel grid computing. Afterwards, the full results including parameters for imputation quality can be downloaded by the cohort. The quality of imputation depends on several features of the input data and on the quality of the haplotype structure in the reference genome. The trio design with medium sequence coverage enables us to establish an accurately haplotyped Dutch reference genome, as the presence of Mendelian transmissions of variants provided an extra level of quality control and aided in the correct estimation of both genotypes and haplotypes.

The GoNL trio design allows calling a substantial amount of *de novo* variation (both SNPs and structural variants). It was recently shown that there is considerable variation in mutation rates within and between families, which in part can be attributed to the age of the father at the time of conception.^28^ With a cohort of 250 families, we will be able to further our understanding of mutational processes, in particular as GoNL also includes 19 families with two twin offspring. The twin pairs will allow us to address questions related to the developmental timing of *de novo* mutations and the frequency of germline mosaicism.

The GoNL data can also be used to address questions related to the demographic history of the Dutch as in-depth information on both mitochondrial^29^ DNA and Y-chromosome will become available. Of immediate utility to the Dutch scientific community is the use of GoNL as a reference for clinical and medical-sequencing projects. Several founder mutations have previously been identified among the Dutch^16^ including founder mutations in the *BRCA1/2* (hereditary breast cancer), *LDLR* (dyslipidemia) and *Tau* (dementia) genes. These findings predict that many genes may incorporate region and population-specific variants. The geographical variation observed in our preliminary analyses of substructure corroborates the importance of the original selection of GoNL samples dispersed throughout the Netherlands. The Dutch samples thus far included in multi-center GWAS studies originate from several different places in the Netherlands and may thus optimally benefit from imputation when the reference material properly reflects this provenance.

Within BBMRI-NL, several additional projects aim at harmonization and enrichment of biobanks within the Netherlands at a national scale. These projects include epigenetic studies, RNA sequencing and metabolomics projects.^14^ To fully understand the functional consequences of genetic variation, we have to integrate across these levels of information. The Dutch biobanks also contain a wealth of phenotypic information, which will become instrumental in pinpointing genes and genetic variants in disease. GoNL will be an excellent starting point to enrich the GWAS samples with more functional DNA variation, and with the BBMRI-NL infrastructure there is an excellent basis to further explain the functional consequences of disease-associated variants.

### Data access

GoNL aims to be an open research source for researchers in the Netherlands and the rest of the world. The sequence data will be made available through dbGaP (http://www.ncbi.nlm.nih.gov/gap). Requests for data can be addressed to the steering committee of GoNL (see http://www.nlgenome.nl).

## Figures and Tables

**Table 1:** Participants by province of Netherlands, and % of the population in that region

*Province*	*Size of population relative to total (% to total of 15–65 years)*	*Number of subjects included in GoNL*	*% of the GoNL sample*	*Number of twin pairs*
Drenthe	2.8	56	7.3	
Friesland	3.8	62	8.1	
Gelderland	11.9	57	7.4	4 MZ, 2 DZ
Groningen	3.5	57	7.4	
Limburg	6.7	58	7.5	1 MZ, 2 DZ
North-Brabant	14.8	68	8.8	1 MZ
North-Holland	16.5	91	11.8	1 MZ
Overijssel	6.7	58	7.5	2 MZ, 2 DZ
Utrecht	9.5	48	6.2	2 MZ
Zeeland	2.2	46	6.0	2 DZ
South-Holland	21.5	168	21.8	
Total		769		19 Twin pairs

Abbreviations: MZ monozygotic, DZ dizygotic.

**Table 2:** Demographic characteristics and basic phenotypes for the GoNL participants. Values in brackets indicate ranges

	*Birth year*	*Average age at sampling (year)*	*Average height (cm)*	*Average BMI (kg/m*^*2*^)	*Average TC level (mmol/l)*	*Average HDL level (mmol/l)*	*Average LDL level (mmol/l)*	*Average TG level (mmol/l)*
Fathers (*N*=250)	1910–64	63.8 (46–87)	178 (160–198)	26.8 (18.1–39.6)	5.42 (2.98–8.23)	1.24 (0.60–2.30)	3.48 (1.29–5.70)	1.56 (0.10–4.80)
Mothers (*N*=250)	1910–64	61.7 (43–86)	166 (145–182)	27.0 (18.6–38.9)	5.67 (3.10–8.70)	1.51 (0.55–2.46)	3.54 (1.48–6.40)	1.35 (0.16–4.65)
Sons (*N*=105)	1945–89	36.1 (20–59)	183 (167–200)	25.1 (17.8–36.6)	4.86 (2.59–7.20)	1.17 (0.61–1.82)	3.07 (0.83–5.35)	1.44 (0.32–4.58)
Daughters (*N*=163)	1940–94	35.9 (19–58)	171 (156–185)	24.6 (18.1–41.6)	4.74 (2.20–7.61)	1.48 (0.61–2.48)	2.79 (0.70–5.40)	1.09 (0.24–4.50)

**Table 3:** Comparison of the designs of the 1000 Genomes and Genome of the Netherlands projects

	*1000G*	*GoNL*
Source of DNA	Cell lines	Blood
Coverage	3–4 ×	>12 ×
Data generation	Multiple centers/ platforms	BGI (Illumina HiSeq)
Population	Unrelated and multiple populations	One population (Dutch) and trio/twin design
Phenotype information	None	Multiple, prospective
